# Metabolic rates are significantly lower in abyssal Holothuroidea than in shallow-water Holothuroidea

**DOI:** 10.1098/rsos.172162

**Published:** 2018-05-30

**Authors:** Alastair Brown, Chris Hauton, Tanja Stratmann, Andrew Sweetman, Dick van Oevelen, Daniel O. B. Jones

**Affiliations:** 1 Ocean and Earth Science, University of Southampton, National Oceanography Centre Southampton, European Way, Southampton SO14 3ZH, UK; 2 Department of Estuarine and Delta Systems, Royal Netherlands Institute for Sea Research (NIOZ-Yerseke), and Utrecht University, PO Box 140, 4400 AC Yerseke, The Netherlands; 3 The Sir Charles Lyell Centre for Earth and Marine Science and Technology, Heriot-Watt University, Edinburgh EH14 4AS, UK; 4 National Oceanography Centre, University of Southampton Waterfront Campus, European Way, Southampton SO14 3ZH, UK

**Keywords:** ecology, evolution, invertebrate, physiology, respiration

## Abstract

Recent analyses of metabolic rates in fishes, echinoderms, crustaceans and cephalopods have concluded that bathymetric declines in temperature- and mass-normalized metabolic rate do not result from resource-limitation (e.g. oxygen or food/chemical energy), decreasing temperature or increasing hydrostatic pressure. Instead, based on contrasting bathymetric patterns reported in the metabolic rates of visual and non-visual taxa, declining metabolic rate with depth is proposed to result from relaxation of selection for high locomotory capacity in visual predators as light diminishes. Here, we present metabolic rates of Holothuroidea, a non-visual benthic and benthopelagic echinoderm class, determined *in situ* at abyssal depths (greater than 4000 m depth). Mean temperature- and mass-normalized metabolic rate did not differ significantly between shallow-water (less than 200 m depth) and bathyal (200–4000 m depth) holothurians, but was significantly lower in abyssal (greater than 4000 m depth) holothurians than in shallow-water holothurians. These results support the dominance of the visual interactions hypothesis at bathyal depths, but indicate that ecological or evolutionary pressures other than biotic visual interactions contribute to bathymetric variation in holothurian metabolic rates. Multiple nonlinear regression assuming power or exponential models indicates that *in situ* hydrostatic pressure and/or food/chemical energy availability are responsible for variation in holothurian metabolic rates. Consequently, these results have implications for modelling deep-sea energetics and processes.

## 1. Introduction

Accurately constraining *in situ* metabolic rates of deep-sea organisms is essential to understanding global carbon cycling [1–4], which itself underpins global climate modelling [5]. Metabolic rates in the deep sea are widely perceived to be low as a result of the low temperature that prevails at depth [6]. However, analysis of bathymetric variation in metabolic rates in diverse marine taxa (fishes, echinoderms, crustaceans, cephalopods) indicates that temperature- and mass-normalized metabolic rates decline strongly with depth in visual pelagic taxa, but not in non-visual pelagic or benthic taxa ([6–13], but see [14]). The ‘visual-interactions hypothesis’ has been proposed to explain this pattern [15], where high metabolic demand results from positive selection for locomotory capacity among visual pelagic predators inhabiting well-lit oceanic waters, and reduced energy expenditure results from relaxation of this selection pressure when predation becomes increasingly limited by light availability.

The potential for any influence of resource limitation (e.g. food/chemical energy or oxygen) or adaptation to low temperature or high hydrostatic pressure on bathymetric trends in metabolic rate have been considered and rejected [6]. Previous explorations of bathymetric influences on metabolic rate have rarely included metabolic rates from abyssal depths (≥4000 m). Instead, these analyses have been dominated by metabolic rates from less than the maximum depth of light penetration (less than 1000 m) [16], confounding capacity to distinguish environmental influences owing to significant covariance in environmental factors (e.g. hydrostatic pressure, temperature, oxygen, food/chemical energy availability, light availability). Despite the plausibility of conclusions drawn by studies examining bathymetric trends in metabolic rate, previous analyses must be regarded with some caution until additional deep-sea metabolic rates reduce the bias towards shallow-water metabolic rates.

Moreover, the limited metabolic rates reported for deep-sea species are predominantly obtained from experiments conducted at shallow-water pressures, but at least some deep-sea taxa are sensitive to depressurization [17]. While abyssal-adapted organisms demonstrate low-pressure intolerance through mortality (e.g. [18,19]), some bathyal fauna may tolerate recovery from approximately 2000 m (e.g. [20]). Even these apparently low-pressure tolerant bathyal species demonstrate pressure-dependent shifts in metabolic rate. For example, metabolic rate is depressed following depressurization in hydrothermal vent shrimp *Mirocaris fortunata* sampled at 1617 m depth [20]. Pressure-related shifts in *M. fortunata*'s metabolic rate are not transient or overcome by acclimation to surface pressure: metabolic rate in *M. fortunata* declines further with sustained exposure to surface pressure (cf. [20,21]), suggesting continuing acclimation. Further, hyperbaric adaptations in mitochondrial function and density may not be detectable at surface pressure: intraspecific cold adaptations in mitochondrial function and density in the killifish *Fundulus heteroclitus* are not apparent when individuals are acclimated to warm temperatures [22]. Consequently, metabolic rates measured at surface pressure may be unrepresentative of deep-sea *in situ* rates, inhibiting accurate assessment of bathymetric trends in metabolic rate that support the visual interactions hypothesis.

The visual interactions hypothesis predicts that there should be no decline in temperature- and mass-normalized metabolic rate with depth in a taxon that is not influenced by visual interactions [7]. The benthic and benthopelagic holothurian lifestyle is not dependent on visual-locomotor interactions with prey or predators at any depth [10,23]. Consistent with the visual interactions hypothesis, previous exploration of metabolic rate in holothurians reported that holothurian metabolic rate is not depth-dependent [10]. However, only 3 of the 26 metabolic rates were from depths greater than 1300 m, limiting statistical power to identify any bathymetric trend. Therefore, the aim of this study was to measure metabolic rates in abyssal holothurians *in situ*, and thus extend the available holothurian metabolic rates and allow reassessment of bathymetric trends in holothurian metabolic rate. Extending available deep-sea holothurian metabolic rates also increases the capacity to identify potential environmental influences (hydrostatic pressure, oxygen, food/chemical energy availability, light availability) on metabolic rate.

## 2. Methods

### 2.1. Study site

*In situ* respiration rates of abyssal Holothuroidae were determined during the RV *Sonne* Joint Program Initiative Oceans – Ecological Aspects of Deep-Sea Mining cruise to the Peru Basin (SO242-2), between August and September 2015. Deployments were made within undisturbed or reference areas in the DISturbance and reCOLonization (DISCOL) experimental area in the abyssal Peru Basin (see [24]) (table 1). The DISCOL region is a site with low organic matter flux [25] and there was no visual evidence of recent organic matter pulse at the deployment sites.

**Table 1.** Location, environmental data, and calculated *in situ* oxygen consumption rates for holothurian specimens.

		sampling location							
incubation start date	species	latitude (S)	longitude (W)	depth (m)	temperature (°C)	salinity	duration (minutes)	oxygen consumption (µmol h^−1^)	total wet mass (g)
05/09/2015	*Mesothuria* sp. 1	07°04.4512′	088°27.0517′	4178.6	1.84	34.83	6399	1.66	28.6
	*Amperima* sp. 1	07°04.4512′	088°27.0517′	4178.6	1.84	34.83	6399	0.71	78.6
	*Amperima* sp. 2	07°04.4512′	088°27.0517′	4178.6	1.84	34.83	6399	2.03	179.7
12/09/2015	*Benthodytes* sp. 1	07°07.5240′	088°27.0439′	4196.5	1.85	35.04	4318	0.27	20.2
	*Amperima* sp. 2	07°07.5240′	088°27.0439′	4196.5	1.85	35.04	4318	0.17	17.9
	*Benthodytes typica*	07°07.5240′	088°27.0439′	4196.5	1.85	35.04	4318	2.30	122.5
17/09/2015	*Benthodytes* sp. 1	07°04.9715′	088°28.1738′	4188.2	1.85	35.05	4741	0.03	34.8
	*Benthodytes* sp. 2	07°04.9715′	088°28.1738′	4188.2	1.85	35.05	4741	1.29	185.0
	*Benthodytes typica*	07°04.9715′	088°28.1738′	4188.2	1.85	35.05	4741	1.60	131.6
24/09/2015	*Benthodytes typica*	07°04.6854′	088°27.4856′	4191.7	1.85	35.04	4257	0.15	47.9
	*Peniagone* sp. 1	07°04.6854′	088°27.4856′	4191.7	1.85	35.04	4257	0.13	57.1
	*Paelopatides* sp. 1	07°04.6854′	088°27.4856′	4191.7	1.85	35.04	4257	0.63	75.5
	*Benthodytes typica*	07°04.6854′	088°27.4856′	4191.7	1.85	35.04	4257	2.37	205.6

### 2.2. Respirometry equipment

Respiration rates were measured using the benthic incubation chamber system 3 (BICS3) previously described by Hughes *et al*. [10]. In brief, the system comprised two units, with each unit composed of two watertight acrylic respirometry chambers housed within an external aluminium protective frame. Each watertight chamber had a 15.38 l capacity and contained a 6000 m depth-rated oxygen optode (Oxygen Optode 3975; Aanderaa, Norway) to continuously measure oxygen concentration (µmol l^−1^). The optode was connected to an RBR XR-420CTDm logger, which also measured and logged conductivity (mS cm^−1^), temperature (°C) and hydrostatic pressure (dbar). Each chamber contained a KUM K/MT 111 motor-driven stirrer. The system was deployed on a GEOMAR ROV elevator platform. The GEOMAR ROV *Kiel 6000* was used to collect individual holothurian specimens from the sediment using a suction sampler. Holothurians were retained at the front of the suction hose during transport to the BICS3. Upon arrival, the holothurian specimen was gently deposited into one of the respirometry chambers, after which the lid was closed to seal the chamber.

### 2.3. Respirometry measurements

Four deployments, each of four respirometry chambers, resulted in oxygen consumption rates from 13 individual holothurians of eight putative species (two *Amperima* spp., two *Benthodytes* spp. and *Benthodytes typica*, *Mesothuria* sp., *Peniagone* sp. and *Paelopatides* sp.) and three background seawater-only incubations. To reduce potential effects from stress associated with collection and transfer to the respirometer, the rate of oxygen consumption was assessed over a period of at least 70 h, beginning 24 h after closure of the last respirometry chamber and ceasing at the commencement of elevator platform recovery. Holothurians were collected intact aboard, and body length and width were measured. Samples were stored at −20°C for subsequent biomass analysis.

Oxygen saturation within respirometry chambers decreased by less than 4% and oxygen depletion was approximately linear (*p* < 0.05, *r^2^* ≥ 0.855) suggesting that holothurian oxygen consumption rates were not affected by oxygen depletion within the respirometry chambers. Oxygen saturation within the background chambers decreased by 0.4% ± 0.1 (mean ± s.d.). Probes were tested for drift subsequent to deployments and no drift was observed. The oxygen consumption of each individual holothurian (*R*, µmol O_2_ h^−1^) (table 1) was calculated from the mean rate of decrease in oxygen concentration within the holothurian respirometry chamber seawater (ΔO_2,H_, µmol O_2_ l^−1^ h^−1^), the mean rate of decrease in oxygen concentration within the background chambers (ΔO_2,B_, µmol O_2_ l^−1^ h^−1^), the volume of the respirometry chamber (*V*_C_, l), and the volume of the holothurian (*V*_H_, l) as:

$$R=\Delta {\text{O}}_{2,H}\cdot (V_{C}-V_{H})-\Delta {\text{O}}_{2,B}\cdot (V_{C}-V_{H}).$$

### 2.4. Comparative metabolic rates

To complement the unique respiration rates of abyssal holothurians with respiration rates from shallower areas, published comparative metabolic rates were compiled using an adaptation of Hughes *et al*. [10] criteria:

1. Metabolic measurements were made using post-larval holothurians, minimizing variation in metabolic rates owing to ontogenetic effects;
2. Holothurians were acclimated to a specified experimental temperature that fell within the temperature range experienced by the species in its natural habitat;
3. Holothurian individual oxygen consumption rates were available either directly, or were possible to derive by calculation;
4. Holothurian masses were provided as wet mass.

Physiological activities such as locomotion or feeding may significantly affect metabolic rate. Consequently, metabolic rates of non-growing, resting, post-absorptive animals (standard metabolic rate) are distinguished from metabolic rates of organisms engaging in routine activity (routine metabolic rate) [26]. The published literature typically did not discriminate between standard metabolic rate and routine metabolic rate, therefore the comparative metabolic rates were interpreted as representing routine metabolic rates to facilitate comparison with the routine metabolic rates reported in this study [10]. Adopting this assumption may be a relatively conservative approach: deep-sea holothurians were not starved prior to *in situ* measurements and reported metabolic rates are therefore routine metabolic rates. Employing routine metabolic rates, which exceed standard metabolic rates, may decrease rather than increase any bathymetric decline. When metabolic rates were reported for a range of wet masses, the mean metabolic rate was determined for size categories 0.1 to <1 g, 1 to <10 g, 10 to <100 g, 100 to <500 g, 500 to <1000 g and 1000 to <1500 g, following Hughes *et al*. [10].

Holothurian collection depth (*CD*) was used to assess the influence of depth on metabolic rate. Holothurians collected intertidally were assigned a *CD* of 10 m as a conservative approach to avoid distortions in using logarithm-transformed depths in regressions [7]. Holothurians collected by diver were assigned a *CD* of 10 m, when *CD* was not reported. Where a depth range was reported in a publication, *CD* was taken as the mid-depth.

### 2.5. Metabolic rate temperature- and mass-normalization

Comparative metabolic rates were measured at experimental temperatures ranging from −1.2°C to 27°C, with total wet mass ranging from 0.6 to 1260.0 g. All metabolic rates were temperature- and mass-normalized to isolate depth-dependence from these confounding factors. Metabolic rates were temperature- and mass-normalized as described below, using temperature- and mass-dependence relationships determined from shallow-water holothurians to avoid incorporating any depth effects within the normalization.

**Figure 1.**
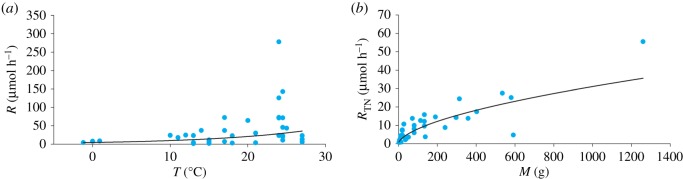
(*a*) Shallow-water holothurian metabolic rate (*R*) as a function of temperature (*T*). Shallow-water holothurian metabolic rate increases significantly with increasing temperature assuming an exponential function (solid line: *R* = 4.702 · e^0.0750*T*^; *F*_1,36_ = 8.796, *p* = 0.005, *r*^2^ = 0.196). (*b*) Shallow-water holothurian temperature-normalized metabolic rate (*R*_TN_) as a function of mass (*M*). Metabolic rate data were normalized to a temperature of 2.5°C using a shallow-water-holothurian-derived *Q*_10_ of 2.12. Shallow-water holothurian metabolic rate increases with increasing mass assuming a power function (solid line: *R*_TN_ = 0.5436 · *M*^0.5858^; *F*_1,36_ = 103.000, *p* < 0.001, *r*^2^ = 0.741). Data from [13,28–41].

Metabolic rate temperature-scaling is described by the exponential function *R* = *f*e^*gT*^, where *f* is a size-independent normalization constant, and *g* is a scaling coefficient that represents the gradient of the linear relationship between temperature (*T*, °C) and *R* following natural logarithmic transformation of *R* [27]. Metabolic rate temperature-dependence was therefore assessed by linear regression of temperature and natural log-transformed metabolic rate. Shallow-water holothurian metabolic rate depended on temperature across a temperature range of −1.2°C to 27°C (figure 1): *R* = 4.702 · e^0.0750*T*^ (*F*_1,36_ = 8.796, *p* = 0.005, *r*^2^ = 0.196, *n* = 38). Consequently, all comparative metabolic rates were temperature-normalized using a *Q*_10_ adjustment:

$$R_{\mathrm{TN}}=R\cdot {Q_{10}}_{N}^{(T-T)/10},$$

where *R*_TN_ is the temperature-normalized metabolic rate (µmol O_2_ h^−1^) at the normalization temperature (*T*_N_, °C). The *Q*_10_ value of 2.12 (*g* = 0.075) derived from the shallow-water holothurian comparative metabolic rates was similar to the mean echinoderm *Q*_10_ value of 2.15 (*g* = 0.077) derived by Hughes *et al*. [10]. All metabolic rates were normalized to 2.5°C (approximating the 2.63°C median temperature of deep-sea holothurian metabolic rate assessments) to reduce the potential for artefacts arising owing to differences in the temperature sensitivity of metabolic rates in shallow-water and deep-sea taxa.

Metabolic rate mass-scaling is described by the power function *R* = *jM*^*k*^ where *j* is a mass-independent normalization constant, and *k* is a scaling coefficient that represents the gradient of the linear relationship between mass (*M*, g) and *R* following logarithmic transformation [27,42]. Metabolic rate mass-dependence was therefore assessed by linear regression of log-transformed *M* and *R*_TN_. Shallow-water holothurian *R*_TN_ depended on mass (figure 1): *R*_TN_ = 0.5436 · *M*^0.5858^ (*F*_1,36_ = 103.000, *p* < 0.001, *r*^2^ = 0.741, *n* = 38). Consequently, all comparative *R*_TN_ were mass-normalized using a mass adjustment:

$$\text{log}R_{\mathrm{TMN}}=\text{ log}R_{\mathrm{TN}}+k(\text{log}M_{N}-\text{log}M),$$

where *R*_TMN_ is the temperature- and mass-normalized metabolic rate at the normalization temperature (*T*_N_, °C) and mass (*M*_N_, g). The *k* value (0.5858) derived from the shallow-water holothurian comparative metabolic rates was used. All metabolic rates were normalized to 70 g (approximating the 66.3 g median mass of deep-sea holothurian metabolic rate assessments) to reduce the potential for artefacts arising from differences in the mass-dependence of metabolic rates in shallow-water and deep-sea taxa. Subsequently, species-specific mean metabolic rates were calculated.

### 2.6. Metabolic rate depth-dependence

Statistical differences between *R*_TMN_ in shallow-water (less than 200 m depth), bathyal (200–4000 m depth), and abyssal (greater than 4000 m depth) holothurians were assessed using Kruskal–Wallis one-way analysis of variance by ranks.

### 2.7. Distinguishing environmental factors contributing to variation in metabolic rate

Multiple environmental factors covary with depth, such as *in situ* hydrostatic pressure (*H*, MPa), annual mean oxygen concentration (*O*_2_, µmol l^−1^), monthly mean food availability (*F*, mg C_org_ m^−2^ d^−1^) and daily peak light availability (*L*, W m^−2^). Data for both environmental factors and biological responses are typically few and consequently distinguishing the relative contributions of environmental factors to any bathymetric variation is challenging and seldom attempted (e.g. [6]).

The potential contributions of environmental factors (*H*, *O*_2_, *F* and *L*) to variation in metabolic rate were initially explored independently, through linear regression and nonlinear regression assuming exponential (*u*e^*vP*^, where *u* is a normalization constant, *v* is a scaling coefficient and *P* is the environmental factor) and power (*wP*^*z*^, where *w* is a normalization constant, *z* is a scaling coefficient and *P* is the environmental factor) functions. Exponential and power functions were selected based on visual inspection of the metabolic rates since there was no preference for model selection based on physiological theory. Nonlinear regressions were assessed by linear regression of factors and natural log-transformed *R*_TMN_ (exponential function), or by linear regression of log-transformed factors and log-transformed *R*_TMN_ (power function). Where environmental data for the collection site were unavailable, environmental data were modelled.

Hydrostatic pressure (*H*) is described by the function:

H=ρ⋅g⋅h,

where *ρ* is the fluid density, *g* is gravitational acceleration and *h* is the fluid height/depth (m). Both *ρ* and *g* vary minimally and *H* therefore depends predominantly on depth. Depth is typically derived from *H* and therefore these parameters were effectively indistinguishable.

*O*_2_ was estimated using the statistical mean oxygen concentration determined by the National Oceanographic Data Center (NODC) climatological analysis and described in the *World Ocean Atlas* 2013 [43]. The NODC climatological analysis is prepared on a one-degree grid and at 102 depth levels between the surface and 5500 m depth. Where mean oxygen concentration was unavailable for the appropriate depth at the holothurian collection location, mean oxygen concentration for the appropriate depth in closest geographical proximity was used.

*F* at depths within the export zone was estimated using the statistical mean net primary production (NPP) determined by constructing a 12-year (2003 to 2014) monthly climatology using the ocean productivity database (www.science.oregonstate.edu/ocean.productivity), which is derived from *MODIS* satellite data based on the standard vertically generalized production model [44]. The ocean productivity database is prepared on a one-sixth-degree grid. Where NPP was unavailable for the appropriate holothurian collection location, NPP in closest geographical proximity was used. Export zone depth was estimated following Lutz *et al*. [45]. Mean monthly *F* at depths below the export zone was estimated from the NPP climatology using particulate organic carbon (POC) flux to the seafloor derived using Lutz *et al*.'s [45] model. Sea surface temperature (SST) was estimated using statistical mean temperature determined by the NODC climatological analysis and described in the *World Ocean Atlas* 2013 [46], at the approximate holothurian collection location and depth. Lateral transfer typically enhances POC flux in submarine canyons relative to adjacent open slopes [47–49]. For example, organic matter availability is 5–30 times higher in the tidal Western Iberian Margin Nazaré canyon than on the open slope [50]. Elevated organic matter has significant ecological impacts on canyon benthic communities (e.g. [51]). Consequently, *F* at holothurian collection locations within submarine canyons was estimated by multiplying the POC flux derived using Lutz *et al*.'s [45] model by 17.5 (the mid-range value for enhanced organic matter availability in canyons presented by García *et al*. [50]). The potential effect of multiple components of *F* (maximum, minimum, mean and standard deviation) were explored.

*L* was estimated using maximum insolation at each holothurian collection location calculated according to Lumb [52] using the solar vector [53], and exponential depth-decay functions presented for coastal and oceanic waters by Lalli & Parsons [54], while assuming no light penetrates to depths below 1000 m [16]. The potential effect of multiple components of *L* (maximum, minimum and mean) were explored.

Subsequently, the potential effect of environmental factors was explored together, through multiple nonlinear regression assuming exponential and power functions, using the components of *F* and *L* with strongest explanatory power (*r^2^*) (*F*_MAX_ and *L*_MAX_).

### 2.8. Sensitivity analysis

Statistical sensitivity to temperature- and mass-scaling parameters (*g* and *k*) was assessed using alternative values employed by Hughes *et al*. [10] (respectively, 0.077 and 0.81). Sensitivity to hydrostatic pressure derived from capture depth parameter was assessed using the minimum depth of occurrence approach with values derived from OBIS [55]. Sensitivity to the canyon POC flux multiplier was assessed using the lower and upper range values for enhanced organic matter availability in canyons (5 and 30) presented by García *et al*. [50], but also by eliminating the canyon POC flux multiplier. Sensitivity to the differentiated coastal and oceanic light decay model was assessed by deriving *L* using only the coastal model or the oceanic model.

## 3. Results

### 3.1. *In situ* oxygen consumption measurements and comparative metabolic rates

Oxygen consumption measurements made at abyssal depths using 13 specimens from eight putative species (table 1) extended the maximum collection depth of holothurian metabolic rates from 3639.6 to 4196.5 m (electronic supplementary material, table S1). A further 22 additional holothurian metabolic rates from 10 species were identified from the literature. Once mean metabolic rate was determined for size categories for each species, 33 additional holothurian metabolic rate data were added to the 26 metabolic rates reported by Hughes *et al*. [10], increasing the number of species with reported metabolic rates from 17 to 35 [10,13,28–41,56–59].

### 3.2. Metabolic rate depth-dependence

**Figure 2.**
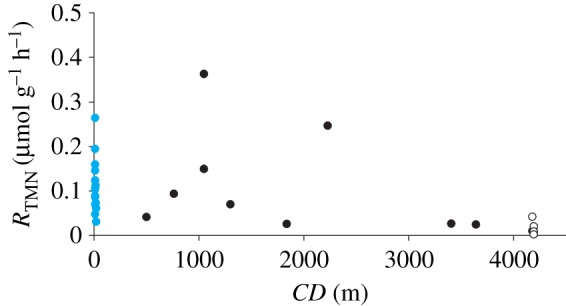
Temperature- and mass-normalized holothurian metabolic rate (*R*_TMN_) over collection depth (*CD*). Metabolic rate data were normalized to a temperature of 2.5°C using a shallow-water-holothurian-derived *Q*_10_ of 2.12, and to a standard total wet mass (*M*) of 70 g using a shallow-water-holothurian-derived mass-scaling coefficient of 0.5858. Blue circles represent shallow-water data (from [13,28–41]), black circles represent deep-sea data (from [10,13,56–59]) and open circles represent data from this study.

*R*_TMN_ did not differ significantly between shallow-water (less than 200 m depth) and bathyal (200–4000 m depth) holothurians, but was significantly lower in abyssal holothurians (greater than 4000 m depth) than in shallow-water holothurians (*F*_2,32_ = 5.808, *p* < 0.007) (figure 2).

### 3.3. Distinguishing environmental factors contributing to variation in metabolic rate

**Figure 3.**
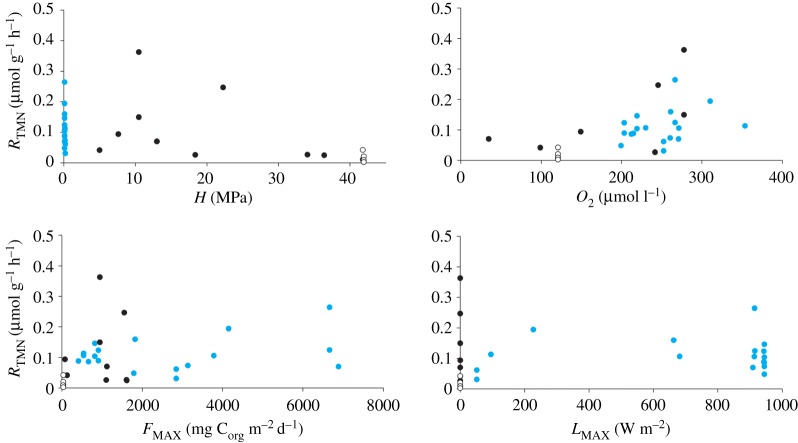
Temperature- and mass-normalized holothurian metabolic rate (*R*_TMN_) as a function of *in situ* hydrostatic pressure (*H*), oxygen concentration (*O*_2_), food availability (*F*_MAX_) or light availability (*L*_MAX_). Metabolic rate data were normalized to a temperature of 2.5°C using a shallow-water-holothurian-derived *Q*_10_ of 2.12, and to a standard total wet mass (*M*) of 70 g using a shallow-water-holothurian-derived mass-scaling coefficient of 0.5858. Blue circles represent shallow-water data (from [13,28–41]), black circles represent deep-sea data (from [10,13,56–59]) and open circles represent data from this study.

Multiple nonlinear regression assuming an exponential function indicated that *H*, *O*_2_, *F*_MAX_ and *L*_MAX_ were not collinear (variance inflation factor < 5.0) and that the exponential function incorporating *H*, *O*_2_, *F*_MAX_ and *L*_MAX_ explained 66.3% of the variation in holothurian *R*_TMN_ (*F*_4,30_ = 14.724, *p* < 0.001, *r*^2^ = 0.663, *n* = 35), but also indicated that only *H* significantly affected *R*_TMN_ (*H p* < 0.001, *O*_2_ *p* = 0.050, *F*_MAX_ *p* = 0.848, *L*_MAX_ *p* = 0.518) (figure 3) (table 2). Nonlinear regression assuming an exponential function indicated that *H* explained 60.6% of the variation in holothurian *R*_TMN_ (*F*_1,33_ = 50.655, *p* < 0.001, *r*^2^ = 0.606, *n* = 35).

**Table 2.** Sensitivity analysis of statistical significance of factors (*H* = hydrostatic pressure; *F*_MAX_ = maximum mean monthly food availability; *O*_2_ = annual mean oxygen concentration; NS = non-significant) affecting variation in holothurian metabolic rate depending on model selection and parametrization. Parameters selected for initial analysis are highlighted in bold.

	parameter				
model	temperature scaling (*g*)	mass scaling (*k*)	hydrostatic pressure	canyon POC flux multiplier	light depth- decay function
	**0.075**/0.077	**0.5858**/0.81	**capture depth**/minimum depth of occurrence	1/5/**17.5**/30	coastal/**coastal & oceanic**/oceanic
exponential	***H***/*H*	***H*/***H*	***H***/*H* and *O*_2_	*H* and *O*_2_/*H* and *O*_2_**/*H***/*H*	*H***/*H***/*H*
power	***F***_**MAX**_/*F*_MAX_	***F***_**MAX**_/*F*_MAX_	***F***_**MAX**_/*H* and *F*_MAX_	NS/NS/***F***_**MAX**_/*F*_MAX_	*F*_MAX_/***F***_**MAX**_/*F*_MAX_

In contrast, multiple nonlinear regression assuming a power function indicated that *H* and *L*_MAX_ were collinear (variance inflation factor > 5.0). Consequently, multiple nonlinear regressions assuming a power function were performed with either *H* or *L*_MAX_ removed. Multiple nonlinear regression assuming a power function indicated that *H*, *O*_2_ and *F*_MAX_ were not collinear (variance inflation factor < 5.0) and that the power function incorporating *H*, *O*_2_ and *F*_MAX_ explained 54.2% of the variation in holothurian *R*_TMN_ (*F*_3,31_ = 12.233, *p* < 0.001, *r*^2^ = 0.542, *n* = 35), but also indicated that only *F*_MAX_ significantly affected *R*_TMN_ (*H p* = 0.234, *O*_2_ *p* = 0.344, *F*_MAX_ *p* = 0.011) (figure 3) (table 2). Similarly, multiple nonlinear regression assuming a power function indicated that *O*_2_, *F*_MAX_ and *L*_MAX_ were not collinear (variance inflation factor < 5.0) and that the power function incorporating *O*_2_, *F*_MAX_ and *L*_MAX_ explained 52.6% of the variation in holothurian *R*_TMN_ (*F*_3,31_ = 11.486, *p* < 0.001, *r*^2^ = 0.526, *n* = 35), but indicated that only *F*_MAX_ significantly affected *R*_TMN_ (*O*_2_ *p* = 0.266, *F*_MAX_ *p* = 0.004, *L*_MAX_ *p* = 0.533) (figure 3) (table 2). Nonlinear regression assuming a power function indicated that *F*_MAX_ explained 48.8% of the variation in holothurian *R*_TMN_ (*F*_1,33_ = 31.435, *p* < 0.001, *r*^2^ = 0.488, *n* = 35).

### 3.4. Sensitivity analysis

Statistical analysis was predominantly robust to alternative values for parameters (table 2). Statistical analysis was robust to the alternative *g* value for temperature-scaling, regardless of the descriptive function assumed. Similarly, statistical analysis was robust to the alternative *k* value for mass-scaling, regardless of the descriptive function assumed. Statistical analysis employing the alternative depth parameter indicated that *O*_2_ significantly affected *R*_TMN_ as well as *H*, assuming an exponential function, and that *H* significantly affected *R*_TMN_ as well as *F*_MAX_ assuming a power function. Statistical analysis was robust to increasing the canyon POC flux multiplier. However, statistical analysis employing decreased canyon POC flux multipliers indicated that *O*_2_ significantly affected *R*_TMN_ as well as *H*, assuming an exponential function. Further, statistical analysis employing decreased canyon POC flux multipliers indicated that no factors significantly affected *R*_TMN_ assuming a power function. Statistical analysis was robust to the light decay model, regardless of the descriptive function assumed.

## 4. Discussion

Metabolic rates of abyssal Holothuroidea were determined *in situ* and synthesized with other available holothurian metabolic rates to explore bathymetric trends in temperature- and mass-normalized metabolic rate. Mean temperature- and mass-normalized metabolic rate did not differ significantly between shallow-water (less than 200 m depth) and bathyal (200–4000 m depth) holothurians, but was significantly lower (82%) in abyssal holothurians (greater than 4000 m depth) than in shallow-water holothurians. Methodological approaches may contribute to this difference. Deep-sea holothurians were not starved prior to *in situ* measurements and the metabolic rates reported in this study are therefore interpreted as routine metabolic rates. Routine metabolic rates are greater than standard metabolic rates, but the published comparative metabolic rates typically did not discriminate between routine and standard metabolic rate. Comparative metabolic rates were therefore interpreted as representing routine metabolic rates to allow statistical analysis. Adopting this assumption is a relatively conservative approach that may diminish bathymetric differences in metabolic rate rather than enhancing them. It is possible that metabolic rates reported in this study represent standard metabolic rates: holothurians may have ceased routine activities in response to sampling and transfer to respirometer chambers. Further, both starting oxygen concentrations (greater than 129 µmol l^−1^) and ending oxygen concentrations (greater than 117 µmol l^−1^) were low, which may have affected metabolic rates: holothurians are typically oxyconformers (e.g. [60]). However, holothurians were observed at different positions within respirometer chambers during the 96 h incubation period and faecal matter was collected from respirometer chambers following incubation, suggesting that holothurians engaged in both movement and digestion. Oxygen depletion was approximately linear (*p* < 0.05, *r^2^* ≥ 0.855) during the ≥70 h of oxygen concentration sampling that followed the 24 h post-sampling recovery period, suggesting both that activity did not differ significantly during the incubation period and that holothurian oxygen consumption rates were not affected by oxygen depletion within the respirometer chambers. Further, while holothurians are typically oxyconformers, they are capable of maintaining relatively high metabolic rate (68% of normoxic metabolic rate) down to oxygen concentrations of approximately 90 µmol l^−1^ [60]. Nonetheless, it is possible that low environmental oxygen concentration contributed to low metabolic rate in abyssal holothurians.

There was notable overlap between the wide-ranging metabolic rates in shallow-water and bathyal deep-sea holothurians (figure 2). Differences in oxygen concentration and food availability may contribute to the dispersion in shallow-water holothurian metabolic rates (figure 3). Similarly, oxygen concentration and food availability may contribute to the elevated metabolic rate reported in *Zygothuria lactea* at 2226 m relative to other deep-sea holothurians. In contrast, the high metabolic rate reported in *Cucumaria frondosa* at 1047.5 m may result from proximity to the species bathymetric range limit. Metabolic rate has been reported to increase significantly in the bathyal lithodid crab *Lithodes maja* as hydrostatic pressure approaches the species 790 m bathymetric range limit [61] and similar responses are expected in other taxa, including holothurians. *C. frondosa* typically occurs at depths shallower than 500 m and there is only a single record of this species occurring deeper than 900 m [55]. Nonetheless, the absence of significant difference between metabolic rate in shallow-water and bathyal holothurians support the dominance of the visual interactions hypothesis at bathyal depths.

In contrast, significantly lower metabolic rate at abyssal depths in this non-visual benthic and benthopelagic echinoderm class suggests that environmental factors contribute to variation in metabolic rates. Although multiple potential factors (oxygen, food/chemical energy, light availability) vary with depth, sufficient holothurian metabolic rates were available to explore the influence of these factors without confounding by collinearity. Statistical exploration of the environmental influences on holothurian metabolic rates indicate that hydrostatic pressure or food/chemical energy availability drive bathymetric variation in holothurian metabolic rate. Which factor is causative of the variation in holothurian metabolic rate depends on the model selected, i.e. exponential or power function, and there is currently no preference for model selection based on physiological theory. The potential influences on metabolic rate of previously considered and rejected environmental factors (hydrostatic pressure or food/chemical energy availability) demand a fundamental reassessment of assumptions regarding environmental influences on metabolic rates in the deep sea. However, unconsidered environmental factors may contribute to differences in metabolic rate between shallow-water and abyssal holothurians. For example, the absence of wave action and hard substratum in abyssal environments may relax the requirement for robust bodies in holothurians which may influence metabolic rate. Further, the analyses presented here represent interspecific comparisons along environmental gradients, precluding any assessment of whether these environmental influences are ecological or evolutionary. Accurately assessing the ecological effects of resource limitation requires intraspecific comparison along an environmental gradient in the absence of intraspecific genetic variation. Accurately assessing adaptation to resource limitation requires intraspecific comparison along an environmental gradient with intraspecific genetic variation, or interspecific comparison along an environmental gradient.

Food/chemical energy may explain 48.8% of variation in metabolic rate among holothurians. There is significant evidence that lower food/chemical energy intake and related activities can result in lower metabolic rate owing to reduced metabolic machinery related to foraging activities and food processing (see [62] and references cited therein). For example, differences in landscape-scale distribution in food between environments (e.g. shallow-water, bathyal, canyon, abyssal) may require differences in foraging strategies and thus in locomotory energy commitment. Such flexibility allows for the possibility of positive selection on metabolic rate driven by food/chemical energy intake and related activities. The influence of chemical energy availability on metabolic rate may mediate the role of chemical energy in driving biodiversity patterns in the deep sea [63,64]. However, hydrostatic pressure offered the strongest explanatory power for the bathymetric variation in metabolic rate in holothurians, potentially explaining 60.6% of metabolic rate variation.

Previous studies have asserted that hydrostatic pressure cannot drive adaptation in metabolic rates (e.g. [6,12]). However, metabolism is a complex process resulting from a network of enzyme-mediated reactions which are affected by hydrostatic pressure [65] and hydrostatic pressure affects metabolic rate in at least some deep-sea species [61]. Although evidence is limited by the relative paucity of studies, fundamental hyperbaric metabolic adaptations have previously been reported in deep-sea bacteria, for example in the cytochrome respiratory system [66,67]. While adaptations in metabolic capacity may allow organisms to moderate hyperbaric impacts [6], such adaptations may also influence metabolic rate. Consequently, it has been proposed that hydrostatic pressure has a critical role in driving bathymetric variation in metabolic adaptations in the deep sea [17,61,68], similar to temperature's role in driving latitudinal patterns in metabolic adaptations (see [69,70]). Oxygen supply influences metabolic rate too (see [62]), offering a mechanism through which environmental oxygen concentration may positively select for reduced metabolic rate.

Significantly lower abyssal metabolic rate in a dominant deep-sea megafauna taxon (abundance and biomass) [16] has implications for modelling of deep-sea processes. For example, estimates of deep-sea respiration and links with community dynamics may be invalid because these estimates assume metabolism does not depend on depth or factors that vary with depth (e.g. [2,12]). Instead, mean temperature- and mass-normalized metabolic rate was 82% lower in abyssal holothurians than in shallow-water holothurians (figure 2). Since deep-sea communities represent a critical component of global biogeochemical cycling [1,3,4,12], erroneous assumptions regarding deep-sea metabolic rates may affect the accuracy of biogeochemical cycle models [5]. Resolving whether hydrostatic pressure or food/chemical energy availability is the dominant driver of bathymetric variation in holothurian metabolic rate in the deep sea, and confirming that temperature does not contribute to depth-related variation, may be achieved by assessing metabolic rates of holothurians present at hadal depths [71] *in situ*. Seafloor sediments in ocean trenches can have higher organic matter concentrations than surrounding abyssal depths [72], but with similar oxygen concentrations [43] and temperatures [46]. Therefore, assessing metabolic rates of hadal holothurians will provide a test of the importance of hydrostatic pressure and food/chemical energy on metabolic rate. Increased metabolic rate in hadal holothurians relative to abyssal holothurians would support the food/chemical energy constraint hypothesis, whereas decreased metabolic rate in hadal holothurians would support the influence of hydrostatic pressure. In contrast, comparable metabolic rate in hadal holothurians would support the oxygen constraint or temperature hypotheses. Such measurements will be key to understanding environmental influences on metabolic rate in the deep sea.

## Supplementary Material

Brown&al. Roy Soc Open Sci ESM Table 1

## Data accessibility

Data are available in table 1 and electronic supplementary material, table S1.
